# Medicine of the Future

**Published:** 1886-08

**Authors:** 


**Medicine of the Future.** An address prepared for the Annual Meeting of the British Medical Association in 1886, by AUSTIN FLINT, Sr., M. D., L. L. D. New York: D. Appleton & Co., 1, 3 and 5 Bond street. 1886.

"The late Dr. Austin Flint was appointed to read the address on Medicine, before the British Medical Association at its meeting in 1886. The manuscript was found among his papers, and the address is printed precisely as it was written. The proof was reverently read by his son, who dedicates this, his father's last literary work, to the profession he so loved and admired." The book contains an excellent portrait of the late Dr. Flint. It is a most fitting memorial volume. The address itself is a most scholarly work, and should be added to the library of every practitioner.

